# Risk of relapse and death from colorectal cancer and its related factors using non-Markovian Multi-State model 

**Published:** 2020

**Authors:** Saeideh Hajebi Khaniki, Vahid Fakoor, Soodabeh Shahid Sales, Habibollah Esmaily, Hamid Heidarian Miri

**Affiliations:** 1 *Student Research Committee, Department of Biostatistics, School of health, Mashhad University of Medical Sciences, Mashhad, Iran*; 2 *Department of Statistics, School of Mathematical Sciences, Ferdowsi University of Mashhad, Mashhad, Iran*; 3 *Cancer research center, Mashhad University of Medical Sciences, Mashhad, Iran*; 4 *Social Determinants of Health Research Center, Department of Biostatistics, Mashhad University of Medical Sciences, Mashhad, Iran *; 5 *Social Determinants of Health Research Center, Department of Epidemiology, Mashhad University of Medical Sciences, Mashhad, Iran *

**Keywords:** Non-Markovian Multi-State Model, Colorectal cancer, Local relapse, Death

## Abstract

**Aim:**

This study aimed at modeling the risk of local relapse and death from colorectal cancer after the first treatment and its related factors using multi-state models.

**Background:**

In cancer studies modeling the course of disease regarding events which happen to patients is of great importance. By considering death as the final endpoint while incorporating the intermediate events, multi-state models have been developed.

**Methods:**

This was a historical cohort study in which 235 patients with colorectal cancer, who referred to Omid Hospital in Mashhad between 2006 and 2011, were studied and followed up until 2017. The transition probabilities to death due to metastasis with or without experiencing local relapse and variables related to them were determined using the non-Markovian multi-state model in three states of disease, local relapse and death.

**Results:**

The probability of not experiencing either of the events, just relapse and death in the first 5 years were 0.45, 0.09 and 0.46 respectively. If patients did not experience any event in the first year of treatment, the probability of relapse and death before the fifth year were 0.04 and 0.33 respectively and if they did experience relapse during this time, the probability of death by the fifth year was 0.62. The stage of cancer was associated with relapse and death, while ethnicity and history of addiction were related to death without relapse and BMI had a significant relationship with death after relapse (p<0.05).

**Conclusion:**

Risk of death in patients with colorectal cancer depends on local relapse and the time between them.

## Introduction

Currently, one of the most common types of cancers throughout the world is colorectal cancer (CRC), which is ranked third after lung and liver cancer (1). In recent decades there has been a significant increase in the incidence of colorectal cancer. The number of new cases of this cancer in the world has increased from 783,000 in 1990 to 1,361,000 in 2012 (2). The World Health Organization(WHO) has also predicted a 77% growth in the detection of new cases of this type of cancer by 2030 (3). Colorectal mortality is roughly equivalent to half of its incidence, and according to the WHO, the number of deaths caused by CRC in 2015 was 774,000 (1).

For appropriate intervention and increasing the survival probability, many studies have been carried out that have led to advances in treatment such as surgery, radiotherapy, chemotherapy, targeted therapy and immunotherapy (4), as well as identification of survival factors such as age, stage or grade of tumor and the type of the first treatment (5). Most of such studies are similar in that the analysis is performed separately for each event (outcome), either relapse or death (6). This way, the correlation between the events would be disregarded. Besides, these approaches don’t give the conditional risk of each event on the other. In addition, the power of statistical tests would be reduced (7).

To handle the preceding considerations, multi-state models are developed which consider death as the final endpoint while incorporating the intermediate events such as local relapse. Indeed, these models suit the situations and states in which patients pass through during the course of follow-up. In other words, multi-state approaches model longitudinal and censored data effectively by regarding dependencies between disease states (8). A multistate model that is more complicated than competing risks is the illness-death model. In the context of progression-free survival, this multistate model would also explore death after progression of disease (6). If the final event that occurs to a patient is just related to the penultimate event, the model would be called Markovian, and if it depends on all other events and the time between them, it is called non-Markovian.

Therefore, the aim of this study was to determine the survival probability and its related factors in patients with colorectal cancer using the non-Markovian illness-death multi-state model. 

## Methods

The data we received was part of a historical cohort study on patients with colorectal cancer who referred to Omid Oncology Hospital, the main referral oncology hospital in northeastern Iran, between 2006 and 2011.The data set had no confidential information; however, the study protocol was approved by the Ethics Committee of the Mashhad University of Medical Sciences (IR.MUMS.REC.1397.127).

The received data set contained demographic and clinical variables. All information was extracted from medical records of patients. Age and sex were recorded from national ID cards. Marital status, history of smoking, addiction and family history of cancer were based on self-report of patients. Body mass index at first visit, was calculated based on weight and height. Moreover, the ethnicity was determined based on self-report and the community to which the person belonged. Type of first treatment (surgery or neoadjuvant chemotherapy) was determined according to dates of treatments in the medical records. Stage of cancer, site of tumor and local relapse were specified based on pathological reports. Death of those patients who passed away in hospital due to metastasis was recorded in medical records. The definitive diagnosis of death due to cancer in those who died outside the hospital was confirmed by death certificate. 

All patients had follow up reviews till the end of 2017 for intermediate event of local relapse or endpoint of death due to metastasis. Those patients whose medical records were not completed or information was not up to date in December 2017, were contacted by staff at the cancer research center of Mashhad university of Medical Sciences. These patients were given hospital appointments for checkups and their status regarding local relapse was ascertained. Patients who survived until the end of the follow-up, withdrew, or died due to other causes were censored. Also, those with metastatic tumors who did not die were censored. 

**Figure 1:**
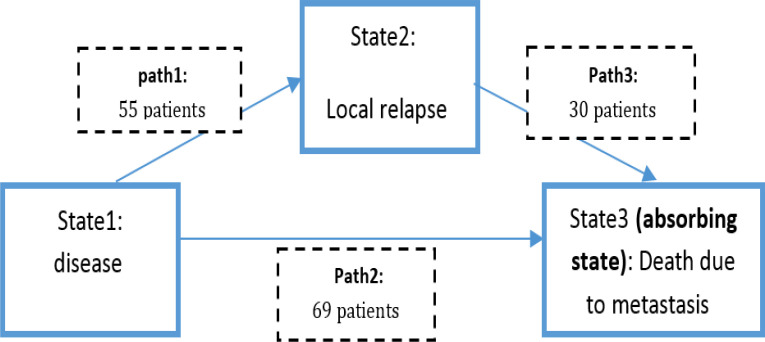
Multi-state model fitted to colorectal cancer data

We used the progressive illness-death model which is a kind of multi-state model that is best suited for situations in which individuals pass through the states only in a forward-moving manner. The word progressive implies that there is no possibility for reverse transition. In case of the present study there are three states as 1-disease 2-local relapse 3-death due to metastasis which forms the three paths ; 1- disease → local relapse 2- disease →death 3- local relapse →death. All patients were in state 1 at the beginning of study. Of those who leave state1, some go to state 2 and some others go directly to state 3 although it is still possible to leave state 2 and go to state 3 (Figure 1). This modeling provides the possibility to estimate the probability transition between states, as well as to identify the hazard ratios of related variables in each path.

The choice of the model to estimate the preceding probabilities depends on whether the future state depends only on the current state (Markov models) or history and time of the transition through states (non-Markovian models) (9). In this study, Markovian assumption can only be assessed in the third path, i.e., local relapse to death, because there is no history for other paths.

There are several methods for assessing this assumption (10-12). In the present study Markovian assumption was assessed using Cox regression model (12)because of the presence of the linear relationship between "time to relapse" and "time to death", high rate of censorship (45.5%), and small sample size (n = 55) in third path. The results of the Cox regression indicated a significant association between "time from disease to local relapse" with the hazard of death in patients who were in state 2. As a result, we fitted the illness-death model adding time to relapse as covariate in the third path and applying non-Markov transition probabilities (9). 

The transition probability for j and h states, and for each two time points as s<t is defined as the probability of being in the state “h” at time “t” conditional on being in state “j” at time “s” considering the history of H_s_. Here the history is “time to local relapse”. In this study, as the Markovian assumption was not held, non-Markov transition probabilities based on nonparametric estimators that were introduced by Alvarez et al. (9) were used which is far different from the Aalen-Johansen estimator (13) that is based on Markov assumption.

We also estimated the state occupation probabilities. It should be noted that this is the same as the transition probability from the initial state (disease) at the beginning of the study (s=0) to the state of local relapse or death. In fact, in this case “s” is fixed to zero and t changes. To put it another way these values are$P_{1h}\left(0,t\right)=P\left(X\left(t\right)=h|X\left(0\right)=\mathrm{disease}\right)$**. **Since these probabilities are transition probabilities from the initial state, we can still use the Aalen-Johansen estimator (14).

The whole disease process of a patient during follow up could be considered as a stochastic process of ${\{X}_{t}:t\geq 0\}$ that t is transition time for every individual. Now the data of i^th^ patient could be considered as a multivariate counting process of $N_{\mathrm{jhi}}\left(t\right),h,j\in \left\{1,2,3\right\},h\neq j,t\leq C_{i}$ which is the number of observed direct transition of j→h in [0,t]. If $Y_{\mathrm{ji}}$ is the number of at risk patients to h state and Z_i_ as covariates, the transition process for$N_{\mathrm{jhi}}(t)$ is defined as$\lambda_{\mathrm{jhi}}\left(t\right){=Y}_{\mathrm{ji}}\left(t\right)\alpha_{\mathrm{jhi}}{(t|Z}_{i}\left(t\right))$ in which αjhit Zi(t))=αjh0(t) exp⁡(∑mβjhmZjhmi(t))(15).

**Figure 2:**
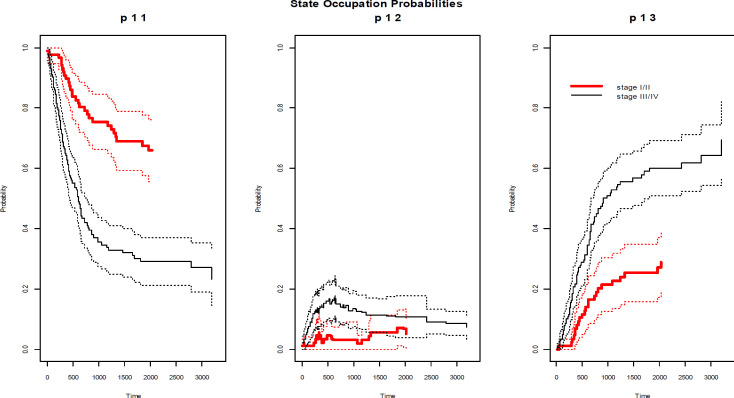
Non-Markov Alvarez et.al state occupation probabilities (solid line) with 95% confidence interval (dotted line) based on stage of disease in course of time (days since first treatment).

Proportional transition hazards model was applied to estimate 3 transition intensities, αjhi(t), between states of disease, local relapse and death in the Continuous-time illness-death model (16) (Fig 1). For instance, α12 represents the instantaneous transition intensity or hazard rate of a transition from state disease to local relapse of colorectal cancer. The model was assumed to follow Cox proportional intensity with heterogeneous baseline intensity functions (i.e., each transition has its own baseline hazard). We emphasize that the model at hand is time inhomogeneous and the process is non-Markov with time-varying transition hazards rather than homogeneous processes with time-constant hazards.

Analysis was performed by R 3.5.3 using survival, TP.idm, etm, mstate and msm packages and p-values less than 0.05 were considered statistically significant. 

## Results

The studied patients consisted of 132 males (56.2%) and 103(43.8%) females. The mean age of the patients was 56.5 ± 14.4 years and 69.4% of them were over the age of 50. Most of the patients were in the normal range (18.5-25 Kg/m2) in terms of BMI, while 17.4% and 25.6% were under and overweight respectively. In 56.5% of patients the location of tumor was the colon and 43.5% in the rectum. First treatment in 57.7% was surgical procedure and 41.3% underwent neoadjuvant chemotherapy as the first therapeutic action. The majority of patients were at stage II or III of disease at the time of the first visit. Most of the patients were married and Fars. 16.6% of the patients had a history of smoking and 6.10% were addicts (Table 1).

**Table 1:** Distribution of variables in terms of local relapse and death due to metastasis.

variable		frequency	local relapse	death before local relapse	death after local relapse
Sex	Male	132(56.2)	32(24.2)	40(40)	17(53.1)
	Female	103(43.8)	23(22.3)	29(36.3)	13(56.5)
Age (Years)	<50	72(30.6)	21(29.2)	15(29.4)	14(66.7)
	>=50	163(69.4)	34(20.9)	54(41.9)	16(47.1)
BMI (Kg/m^2^)	<18.5	41(17.4)	22(9.0)	11(34.4)	5(55.6)
	18.5-25	134(57.0)	36(26.9)	45(45.9)	21(58.3)
	25-30	42(17.9)	5(11.9)	7(18.9)	1(20.0)
	>30	18(7.7)	5(27.8)	6(46.2)	3(60.0)
Site of Tumor	Colon	133(56.5)	37(27.8)	37(38.5)	19(51.4)
	Rectum	102(43.4)	18(17.6)	32(38.1)	11(61.1)
Type of First Treatment	Surgery	138(58.7)	34(24.6)	37(35.6)	16(47.1)
	Neoadjuvant chemotherapy	97(43.1)	21(21.6)	32(42.1)	14(66.7)
Stage of Disease	I	14(6.0)	1(7.1)	1(7.7)	1(100.0)
	II	75(31.9)	13(17.3)	13(21.0)	8(61.5)
	III	87(37.0)	24(27.6)	21(33.3)	15(62.5)
	IV	59(25.1)	17(28.8)	34(81.0)	6(35.3)
Marital Status	Single	6(2.6)	3(50.0)	2(66.7)	2(66.7)
	Married	217(92.3)	49(22.6)	62(36.9)	27(55.1)
	Widow/divorced	12(5.1)	3(25.0)	5(55.6)	1(33.3)
Ethnicity	Fars	201(85.5)	46(22.9)	55(35.5)	26(56.5)
	Non-Fars	34(14.5)	9(26.5)	14(56.0)	4(44.4)
Family History of Cancer	Yes	49(20.9)	15(30.6)	10(29.4)	6(40.0)
	No	186(79.1)	40(21.5)	59(40.4)	24(60.0)
Smoking	Yes	39(16.6)	10(25.6)	11(37.9)	5(50.0)
	No	196(83.4)	45(23.0)	58(38.4)	25(55.6)
Addiction	Yes	25(10.6)	7(28.0)	9(50.0)	6(85.7)
	No	210(89.4)	48(22.9)	60(37.0)	24(50.0)

**Table 2:** Non-Markov Alvarez et.al State occupation probabilities $P_{1h(0,t)}=P(X\left(t\right)=\mathrm{hX}\left(0\right)=\mathrm{disease}))$ and Transition probabilities.

State/ Path	From time (s)	To time (t)	State occupation /Transition probability	95% CI State occupation /Transition probability
Disease	0	1^st^ year	0.74	(0.68-0.79)
	0	2^nd^ year	0.56	(0.50-0.63)
	0	5^th^ year	0.45	(0.38-0.51)
	0	7^th^ year	0.43	(0.37-0.50)
	0	10^th^ year	0.39	(0.31-0.47)
Local relapse	0	1^st^ year	0.10	(0.06-0.14)
	0	2^nd^ year	0.11	(0.06-0.16)
	0	5^th^ year	0.09	(0.04-0.15)
	0	7^th^ year	0.08	(0.05-0.11)
	0	10^th^ year	0.07	(0.04-0.10)
Death	0	1^st^ year	0.15	(0.11-0.21)
	0	2^nd^ year	0.33	(0.26-0.39)
	0	5^th^ year	0.46	(0.39-0.53)
	0	7^th^ year	0.48	(0.41-0.56)
	0	10^th^ year	0.53	(0.43-0.63)
Disease → Local relapse	1^st^ year	10^th^ year	0.04	(0.01-0.07)
	2^nd^ year	10^th^ year	0.02	(0.00-0.05)
	5^th^ year	10^th^ year	0.00	(0.00-0.02)
Disease → Death	1^st^ year	10^th^ year	0.42	(0.31-0.54)
	2^nd^ year	10^th^ year	0.28	(0.14-0.41)
	5^th^ year	10^th^ year	0.12	(0.00-0.26)
Local relapse → Death	1^st^ year 2^nd^ year	10^th^ year 10^th^ year	0.62 0.44	(0.37-0.87) (0.19-0.69)

**Table 3:** Multiple Proportional transition hazards models.

Path	Variable	β	HR	Z statistic	*P-value*
Disease → Local relapse	Site of tumor^1^: Rectum	-0.37	0.69	-1.24	0.214
	Stage^2^: I/II	-0.79	0.45	-2.44	0.014
	Marital status^3^:married	-0.59	0.55	-0.95	0.344
	Divorced / widow	-0.47	0.63	-0.55	0.582
	Family history of cancer^4^: No	-0.29	0.75	-0.89	0.376
	Addiction^5^: No	-0.19	0.82	-0.44	0.659
	Age^6^: >50 years	-0.18	0.83	-0.62	0.538
	BMI^7 (^kg/m^2)^:18.5-25	0.17	1.18	0.44	0.658
	25-30	-0.60	0.55	-1.05	0.293
	>30	0.21	1.24	0.37	0.711
Disease → Death due to metastasis	Type of first treatment^8^: neoadjuvant chemotherapy	0.24	1.28	0.95	0.344
	Stage^2^: I/II	-1.44	0.24	-4.56	<0.001
	Ethnicity^9^: Non-Fars	0.99	2.71	3.06	0.002
	Family history of cancer^4^: No	0.54	1.72	1.53	0.127
	Addiction^5^: No	-0.52	0.59	-1.38	0.168
	Age^6^: >50 years	0.76	2.15	2.46	0.013
	BMI^7(^kg/m^2)^:18.5-25	0.13	1.14	0.39	0.698
	25-30	-0.77	0.46	-1.51	0.131
	>30	0.23	1.25	0.43	0.668
Local relapse → Death due to metastasis	Type of first treatment^8^: neoadjuvant chemotherapy	0.81	2.25	1.81	0.070
	Stage^2^: I/II	0.68	1.98	1.33	0.182
	BMI^7(^kg/m^2)^: 18.5-25	-1.16	0.31	-1.97	0.048
	25-30	-2.67	0.07	-2.28	0.022
	>30	-1.77	0.17	-2.14	0.032

**Reference categories:** ^1^ colon, ^2^ III/IV, ^3^ single ^4^ yes, ^5 ^yes, ^6^ <=50, ^7^ <18.5 kg/m^2 ^, ^8^ surgery , ^9^ Fars

Out of the 235 patients in state 1, 55 went to state 2 (locally relapsed) from which 30 went to state 3 (death due to metastasis). Also 69 patients went directly to state3 and 111 patients were censored from the whole process; in other words they did not experience local relapse and also they survive till the end of study. 23% of patients experienced local relapse with a median time of 295 days after first treatment. 

The frequency of death due to colorectal cancer was 99(42.1%) with a median time of 544 days after first treatment. Of those who passed away, 30 patients had local relapse of whom the median time of survival was 483 days which was quite different from those who had not (676 days).The occupation probabilities are given for different “t” in Table 2. It is bserved that the probability of staying in the initial state (neither local relapse nor death) gradually decreased over follow up time, but the slope of this decline was much higher in the first 3 years. This decrease was from 0.45 at the end of the fifth year to 0.39 at the end of the follow-up period (10th year). On the other hand, the local relapse occupation probability increased and reached 0.11 in the second year, whereas its downward trend after the second year was due to the fact that this state was transient. Also, probability of being in death state as an absorbing state was augmented as time passed. In the middle of the follow-up (5^th^ year), the patient's probability of death was 0.46, reaching 0.33 in the 10^th^ year.

According to the estimated values of transition probabilities (Table 2), if the patient did not have local relapse by the first year, i.e. remaining in state 1, the probability of relapse but not dying (being in state 2) would be 0.04 at the end of follow up, although it would be 0.02 provided that the patient remained in state 1 for two years. Furthermore, if the patient had not gone to relapsed status until the fifth year, the risk of recurrence afterwards would be zero. In addition, risk of death at the end of follow up, would be 0.42, 0.28 and 0.12 in those who had not experienced local relapse till first, second and fifth year after first treatment respectively. On the other hand, if the patient had local relapse during the first year, he would have passed away, with a probability of 0.9. Estimated state occupation and transition probabilities based on stage of disease are illustrated in Figures 2.

To determine the significant variables in each path of the illness-death model, all variables were entered in a separate univariate model in each path and variables with p-values less than 0.25 entered the multiple transition hazard models. As the results of Table 3 illustrate, in the first path, i.e., disease to local relapse, the stage of the disease was significant. The hazard of relapse in patients who were in stage I or II was 0.45 times more than that of patients in stage III or IV. Age group and ethnicity were associated with transition of the patient to the state of death. Moreover, with the decrease in the stage of CRC, the hazard of death without relapse was reduced (HR=0.24). On the other hand, the hazard of death without recurrence in patients whose ethnic origin was non-Fars, was 2.71 times more that of the patients who were Fars. The hazard ratio of death without recurrence in patients over 50 to below 50 years was 2.15. Also BMI was associated with occurrence of death after relapse. The hazards of death in the third path for patients with normal BMI, overweight (BMI 25-30 kg/m2) or obese were 0.31, 0.069 and 0.17 times that of patients with a BMI of less than 18.5 kg/m2.

## Discussion

This study was conducted to determine the risk of local relapse and death from colorectal cancer and its related factors using non-Markovian illness-death model in which modeling of colorectal cancer is divided into three parts including disease to local relapse, disease to death without relapse and relapse to death. In the path of disease to local relapse, relapse is considered as the final event. In the present study, the stage of the disease was significant in this path. This means that patients who were in stage I or II of colorectal cancer at the first visit had lower hazard of relapse than those in stage III or IV. In some studies, using Cox models, the disease stage was identified as one of the factors associated with local relapse of colorectal cancer (17, 18), which is consistent with the results of the current study. However, in some others, the stage of the disease is not one of the factors influencing the relapse of the disease (19), which may be due to the different definition of the variable of the disease stage using three separate variables of tumor, node and metastases instead of using a TNM staging system as was employed in ours In addition, it should be noted that the association of the stage of the disease to local relapses in colorectal cancer had been shown in other studies with multi-state approach (20-23). Moreover, in one of the studies conducted with multi-state method, age (with a cut point of 75 years) was associated with relapse of the disease (21), which contradicted the results of this study. In this study, age classification was performed based on the onset of colorectal cancer screening.

In the present study, disease stage, age, and ethnicity were associated with death without relapse and BMI was associated with the death after relapse. In studies based on semi-parametric Cox models, tumor size, metastasis, body mass index, marital status, tumor grade, history of addiction, recurrence, stage of disease and obstruction were reported as factors associated with survival from colorectal cancer (24-31). In some studies, based on the Markov approach, gender and site of the lesion were also associated with death after relapse (20-23). One important reason for obtaining different results could be applying the distinct modeling, not regarding relapse as an intermediate event or not assessing Markov assumption. However, considering the clinical point of view, time to local relapse of CRC could affect survival of patient (32) and entering it to model would adjust the effects of other covariates. 

One of the features of illness-death model is the feasibility of transition probability between disease, relapse, and reaching the absorbing state of death at any two given time points. In this study, due to the absence of Markov assumption, a non-Markovian method was used to evaluate these transition probabilities. Regarding the estimated non-Markovian probabilities, staying in the initial state (the disease) gradually decreased over time. Also, the risk of local relapse or death between the fifth and the tenth follow-up year was 0.04. On the other hand, the probability of staying in transient state of relapse had increased by the second year, indicating that most of the relapse events occurred in the two first years of the first treatment. Overall, these results suggest that most events for a patient suffering from colorectal cancer occurred in the early years of the disease and as time passed the patient's chance of changing the condition was reduced. It was observed that the probability of recurrence of the disease decreased over time, and if the disease has not recurred by the fifth year, the chance of it returning after this time was zero. Furthermore, with the increase in the duration between the first treatment and the disease, the patient's likelihood of death decreased.

One of the limitations of our study was the existence of missing values in some variables which lead to reduction in sample size. Of course, since the loss of data was completely random, it did not cause bias but generally multi-state models require a relatively large sample size due to fitting a separate model in each path. In this study when we assumed the stage of disease as a four category variable (stage I, II, III, IV), the estimates of hazard ratios in the third path were very large as a result of sparse data. So, we had to combine the stages of I and II, III and IV. Nevertheless, performing multiple imputations for missing values and comparing the results by leaving out missing analysis is recommended. In addition, one of the other important issues in almost all cancer studies is the problem of unknown time of onset of disease which causes left truncation. Therefore, having this exact or nearly exact time at hand would help to model the whole process of disease more accurately.

In many cases, the patient's prognosis is affected by intermediate events (such as local relapse in most of cancers). In other words, the prediction of patient survival can be changed over time based on events which occur to a patient. On the other hand, the oncologist’s approach to treat the disease and the factors that affect this process all depend on proper modeling. 

In the current study, the disease stage was associated with the transition of disease from disease to local relapse. Also, the stage of the disease, ethnicity and age were associated with the risk of death without local relapse. The patient's BMI was also associated with the risk of death after local relapse.

## References

[1] WHO Cancer February 2018. http://www.who.int/mediacentre/factsheets/fs297/en/.

[2] Gandomani HS, Aghajani M, Mohammadian-Hafshejani A, Tarazoj AA, Pouyesh V, Salehiniya H (2017). Colorectal cancer in the world: incidence, mortality and risk factors. Biomed Res Ther.

[3] Karsa L, Lignini T, Patnick J, Lambert R, Sauvaget C (2010). The dimensions of the CRC problem. Best Pract Res Clin Gastroenterol.

[4] São Julião GP, Habr-Gama A, Vailati BB, Araujo SEA, Fernandez LM, Perez RO (2017). New Strategies in Rectal Cancer. Surg Clin.

[5] Desantis C, Siegel R, Jemal A (2012). Cancer treatment & survivorship facts & figures 2012-2013. CA Cancer J Clin.

[6] Beyersmann J, Allignol A, Schumacher M (2011). Competing risks and multistate models with R.

[7] Masoudi S, Pourdanesh F, Biglarian A, Rahgozar M (2015). Investigation of the follow up and prognosis of patients with squamous cell carcinoma of the mouth using the Markov multistate model. J Epidemiol.

[8] Geskus RB (2015). Data analysis with competing risks and intermediate states.

[9] de Uña-Álvarez J, Meira-Machado L (2015). Nonparametric estimation of transition probabilities in the non‐Markov illness‐death model: A comparative study. Biometrics.

[10] Jones MP, Crowley J (1992). Nonparametric tests of the Markov model for survival data. Biometrika.

[11] Rodríguez‐Girondo M, de Uña‐Álvarez J (2012). A nonparametric test for Markovianity in the illness‐death model. Stat Med.

[12] Rodríguez‐Girondo M, Uña‐Álvarez Jd (2016). Methods for testing the Markov condition in the illness-death model: a comparative study. Stat Med.

[13] Aalen OO, Johansen S (1978). An empirical transition matrix for non-homogeneous Markov chains based on censored observations. Scand J Stat.

[14] Datta S, Satten GA (2001). Validity of the Aalen–Johansen estimators of stage occupation probabilities and Nelson–Aalen estimators of integrated transition hazards for non-Markov models. Stat Prob Lett.

[15] Klein JP, Van Houwelingen HC, Ibrahim JG, Scheike TH (2016). Handbook of survival analysis.

[16] Gelfand AE, Diggle P, Guttorp P, Fuentes M (2010). Handbook of spatial statistics.

[17] Pietra N, Sarli L, Thenasseril B, Costi R, Sansebastiano G, Peracchia A (1998). Risk factors of local recurrence of colorectal cancer: a multivariate study. Hepatol Gastroenterol.

[18] Abaspour S (2010). The rate and type of relapse of colorectal cancer in patients undergoing surgery referring to Jundishapur Medical Sciences Hospitals in Ahvaz, 997-2007.

[19] Fatemi SR, Pourhoseingholi MA, Asadi F, Vahedi M, Pasha S, Alizadeh L (2015). Recurrence and five-year survival in colorectal cancer patients after surgery. Iran J Cancer Prev.

[20] Gilard-Pioc S, Abrahamowicz M, Mahboubi A, Bouvier A-M, Dejardin O, Huszti E (2015). Multi-state relative survival modelling of colorectal cancer progression and mortality. Cancer Epidemiol.

[21] Dancourt V, Quantin C, Abrahamowicz M, Binquet C, Alioum A, Faivre J (2004). Modeling recurrence in colorectal cancer. J Clin Epidemiol.

[22] Huszti E, Abrahamowicz M, Alioum A, Binquet C, Quantin C (2012). Relative survival multistate Markov model. Stat Med.

[23] Cyvoct C, Quantin C, Broet P, Benhamiche A, Brunet-Lecomte P, D'athis P (1999). Prognostic factors of recurrence and/or death in colorectal cancer: multistate modeling. Rev Epidemiol Sante Publique.

[24] Parsaei R, Fekri N, Shahid Sales S, Afzal aghaei M, Sherbaf A, Esmaily H (2015). Prognostic factors in the survival of patients with colorectal cancer. J North Khorasan Univ Med Sci.

[25] Moghimi, Dehkordi B, Safaei A, Zali Mr (2008). Survival rates and prognostic factors in colorectal cancer patients. Sci J Med.

[26] Chao-Hsien L, Cheng S-C, Hong-Yi T, Chang S-C, Ching C-Y, Shu-Fen W (2018). The Risk Factors Affecting Survival in Colorectal Cancer in Taiwan. Iran J Public Health.

[27] Huang Y, Alzahrani NA, Liauw W, Arrowaili A, Morris DL (2018). Survival difference between mucinous vs non-mucinous colorectal cancer following cytoreductive surgery and intraperitoneal chemotherapy. Int J Hyperthermia.

[28] Prasanna T, Karapetis CS, Roder D, Tie J, Padbury R, Price T (2018). The survival outcome of patients with metastatic colorectal cancer based on the site of metastases and the impact of molecular markers and site of primary cancer on metastatic pattern. Acta Oncologica.

[29] Chiu KW, Lam KO, An H, Cheung GT, Lau JK, Choy TS (2018). Long-term outcomes and recurrence pattern of 18F-FDG PET-CT complete metabolic response in the first-line treatment of metastatic colorectal cancer: a lesion-based and patient-based analysis. BMC Cancer.

[30] Newland RC, Chan C, Chapuis PH, Keshava A, Rickard MJ, Young CJ (2018). Competing risks analysis of the effect of local residual tumour on recurrence and cancer-specific death after resection of colorectal cancer: implications for staging. Pathology.

[31] Yamano T, Yamauchi S, Tsukamoto K, Noda M, Kobayashi M, Hamanaka M (2018). Evaluation of appropriate follow-up after curative surgery for patients with colorectal cancer using time to recurrence and survival after recurrence: a retrospective multicenter study. Oncotarget.

[32] Yazilitas D, Özdemir N, Hocazade C, Demirci NS, Zengin N (2016). The clinical and pathological features affecting the time of relapse in patients with early stage colorectal cancer. J Cancer Res Ther.

